# Protein Arginine Methyltransferase 1 Interacts with and Activates p38α to Facilitate Erythroid Differentiation

**DOI:** 10.1371/journal.pone.0056715

**Published:** 2013-03-06

**Authors:** Wei-Kai Hua, Yuan-I Chang, Chao-Ling Yao, Shiaw-Min Hwang, Chung-Yi Chang, Wey-Jinq Lin

**Affiliations:** 1 Institute of Biopharmaceutical Sciences, National Yang-Ming University, Taipei, Taiwan; 2 Department of Chemical Engineering and Materials Science, Yuan Ze University, Chung-Li, Taiwan; 3 Bioresource Collection and Research Center, Food Industry Research and Development Institute, Hsinchu, Taiwan; 4 Heart Center, Cheng Hsin General Hospital, Taipei, Taiwan; Kinki University School of Pharmaceutical Sciences, Japan

## Abstract

Protein arginine methylation is emerging as a pivotal posttranslational modification involved in regulating various cellular processes; however, its role in erythropoiesis is still elusive. Erythropoiesis generates circulating red blood cells which are vital for body activity. Deficiency in erythroid differentiation causes anemia which compromises the quality of life. Despite extensive studies, the molecular events regulating erythropoiesis are not fully understood. This study showed that the increase in protein arginine methyltransferase 1 (PRMT1) levels, via transfection or protein transduction, significantly promoted erythroid differentiation in the bipotent human K562 cell line as well as in human primary hematopoietic progenitor CD34^+^ cells. PRMT1 expression enhanced the production of hemoglobin and the erythroid surface marker glycophorin A, and also up-regulated several key transcription factors, GATA1, NF-E2 and EKLF, which are critical for lineage-specific differentiation. The shRNA-mediated knockdown of PRMT1 suppressed erythroid differentiation. The methyltransferase activity-deficient PRMT1G80R mutant failed to stimulate differentiation, indicating the requirement of arginine methylation of target proteins. Our results further showed that a specific isoform of p38 MAPK, p38α, promoted erythroid differentiation, whereas p38β did not play a role. The stimulation of erythroid differentiation by PRMT1 was diminished in p38α- but not p38β-knockdown cells. PRMT1 appeared to act upstream of p38α, since expression of p38α still promoted erythroid differentiation in PRMT1-knockdown cells, and expression of PRMT1 enhanced the activation of p38 MAPK. Importantly, we showed for the first time that PRMT1 was associated with p38α in cells by co-immunoprecipitation and that PRMT1 directly methylated p38α in *in vitro* methylation assays. Taken together, our findings unveil a link between PRMT1 and p38α in regulating the erythroid differentiation program and provide evidence suggesting a novel regulatory mechanism for p38α through arginine methylation.

## Introduction

Circulating red blood cells are derived from hematopoietic progenitors through an intricate process of erythropoiesis which is essential for maintaining a vital physiological condition. In addition, stimulating erythropoiesis has a wide application in treating anemia caused by chemotherapy, chronic renal failure and hematological diseases [1]. Despite extensive studies, our understanding of regulatory events involved in erythroid differentiation remains incomplete.

A variety of cytokines acts in concert to regulate erythroid differentiation [2]. Among these, erythropoietin (EPO) is the primary cytokine regulating various stages of erythropoiesis in our body. Intracellular signaling pathways such as the JAK/STAT [3], mitogen-activated protein kinase (MAPK) [4] and PI3 kinase/Akt [5] cascades have been shown to mediate these extracellular signals. Activation of the p38 MAPK pathway contributes critically to the erythroid differentiation of leukemia cell lines induced by various agents [6], [7] and of primary CD34^+^ hematopoietic progenitors induced by EPO [8]. There exist multiple isoforms of p38; however the distinctive role of each isoform in erythropoiesis is still elusive. Moreover, although some downstream effectors of p38 MAPK, such as activating transcription factor-2 (ATF-2) and cyclic AMP response element binding protein (CREB), have been reported to participate in the induction of the γ-globin gene [9], the detailed upstream regulatory events and downstream effectors of the p38 MAPK pathway during erythroid differentiation are yet to be elucidated.

Posttranslational modifications expand the chemical diversity of side chains of amino acids and provide dynamic and reversible modulations of the activity of proteins to meet the need of cellular functions. Protein arginine methylation, which adds one or two methyl groups to the guanidine nitrogen of arginine residues, is emerging as a pivotal posttranslational modification involved in various cellular processes including transcription regulation, DNA repair, RNA processing and signal transduction [10], [11]. Protein arginine methyltransferase 1 (PRMT1) is the first cloned [12] and the predominant PRMT in mammalian cells [13]. PRMT1 can regulate gene expression by methylating transcription factors and histones. The methylation of NIP45 by PRMT1 facilitates its interaction with NF-AT and thus stimulates cytokine gene expression [14]. The transcriptional activity of RUNX1 is potentiated by PRMT1 methylation, which abrogates its association with the repressor SIN3A [15]. PRMT1 can modify histone H4 on Arg3, which permits the subsequent acetylation of histone H3 and thus activates gene expression [16]. In addition, arginine methylation is reported to affect the enzymatic activity of the modified proteins. For example, PRMT1-mediated methylation of the NS3 protein of hepatitis C virus inhibits its helicase activity [17]. Evidence supporting the crosstalk between arginine methylation and phosphorylation is emerging. Arginine methylation of FOXO transcription factors and anti-apoptotic BAD proteins by PRMT1 inhibits their phosphorylation by Akt [18], [19]. PRMT1 transiently methylates estrogen receptor α and triggers its interaction with PI3K and Src tyrosine kinase [20]. The methylation of Axin by PRMT1 enhances its phosphorylation by GSK3β (glycogen synthase kinase 3β), leading to an increased stability [21].

Given the broad range of cellular functions regulated by protein arginine methylation, this study explored the involvement of this posttranslational modification in modulating erythroid differentiation. Our results provide solid evidence for a novel role of PRMT1 in promoting erythroid differentiation and suggest arginine methylation as a novel mechanism for regulating p38α functions.

## Methods

### Erythroid Differentiation

Human chronic myelogenous leukemia K562 cells and human erythroleukamia HEL cells were obtained from BCRC (Bioresource Collection and Research Center, Taiwan). Both cell lines were cultivated in RPMI 1640 medium supplemented with 10% fetal bovine serum (Biological Industries). To induce erythroid differentiation, cells were treated with cytosine arabinoside (araC, 1 µM) or sodium butyrate (NaB, 2 mM for K562 and 0.1 mM for HEL). Hemoglobin production was detected using a benzidine/hydrogen peroxide solution [6]. The dark violet particles of oxidized benzidine were readily distinguished under a light microscope. Two to three hundred cells were examined in each assay. Surface expression of glycophorin A, a characteristic of erythroid cells, was detected by flow cytometry.

### Isolation of CD34^+^ Hematopoietic Progenitor Cells and Induction of Erythroid Differentiation

Isolation and expansion of CD34^+^ cells were performed as described previously [22]. To induce differentiation toward erythroid lineage, the expanded CD34^+^ cells were cultivated (5 × 10^4^ cells/ml) in media without a cytokine cocktail for 6 hr prior to the addition of erythropoietin (EPO; 1 U/ml). Three days later, the cells were again treated with EPO. Erythroid differentiation was examined 7 days after the first EPO treatment by surface expression of glycophorin A and benzidine staining. The TAT-mediated protein transduction system was applied to examine the effect of PRMT1. The purified recombinant TAT-fused HA-PRMT1 and the control TAT-fused HA-GFP proteins were added to the CD34^+^ cells 6 hr before the first EPO treatment and again at the time of the EPO treatment.

### Ethic Statement

The human umbilical cord blood was obtained from Taoyuan General Hospital, Taiwan, with an informed written consent from donors and the prior approval from the ethic committee of Institutional Review Board (IRB). The procedure was conducted following the approved guideline of IRB. The data obtained in this study were analyzed anonymously.

### Flow Cytometric Analysis

Cells were washed, incubated in PBS containing 2% BSA for 30 min at 4°C and stained with anti-human FITC-conjugated glycophorin A antibodies (Abcam; 1 µl in 200 µl of 1% BSA-PBS) for 30 minutes. After washing, cells were suspended in 1 ml of 1% BSA-PBS and subjected to flow cytometric analysis using a FACScan flow cytometer (BD Biosciences) equipped with Cellquest software (BD Biosciences).

### Examination of PRMT1 Activity in CD34^+^ Progenitor Cells

Human primary CD34^+^ hematopoietic progenitor cells were resuspended in extraction buffer (50 mM Tris-HCl, pH 7.4, 1 mM EDTA, 1 mM EGTA, 40 µg/ml leupeptin, 40 µg/ml aprotinin, 20 µg/ml pepstatin, and 1 mM PMSF) and homogenized on ice with a glass tissue grinder. The homogenates were centrifuged at 16,000× *g* for 60 min at 4°C, and supernatants were collected. To examine endogenous PRMT1 activity, cell homogenates (2 µg) were incubated with purified recombinant hnRNP A1, A2 or K proteins (2 µg), which are known substrates for PRMT1 [23], [24], [25], in 25 mM Tris-HCl, pH 8.0, and *S*-adenosyl-L-[*methyl*-^3^H] methionine (^3^H-AdoMet) at 30°C for 30 min. Reactions were stopped by the addition of SDS sample buffer and then subjected to SDS-PAGE. Gels were stained, de-stained and then soaked in the fluorographic enhancer EN^3^HANCE (Perkin Elmer). After dried, the gels were exposed to X-ray films (GE) at −70°C. Methyl incorporation was analyzed by fluorography or by scintillation counting as described previously [23], [24].

### Western Blotting

Cells were lysed in RIPA buffer (150 mM NaCl, 50 mM Tris, pH 7.4, 0.1% SDS, 1% Triton X-100, 1% sodium deoxycholate, 1 mM ethylenediaminetetraacetic acid, 1 mM phenylmethylsulfonyl fluoride, 10 µg/ml aprotinin, 10 µg/ml leupeptin, 10 µg/ml pepstatin, 1 mM sodium fluoride, 1 mM sodium orthovanadate, and 25 mM β-glycerophosphate). Proteins were subjected to Western blot analysis. The primary antibodies used were anti-HA (HA.11, Covance; 1∶1000), anti-PRMT1 (Sigma; 1∶1000), anti-phospho-p38 (Cell Signaling; 1∶500), anti-p38 (Cell Signaling; 1∶1000), anti-p38β (Cell Signaling; 1∶1000), anti-ERK2 (Cell Signaling; 1∶1000), anti-Flag (M5, Sigma; 1∶1000), and anti-GAPDH (Abcam; 1∶10000).

### Real-time Reverse Transcription PCR

Total RNA was extracted using an illustra RNAspin Mini Isolation Kit (GE Healthcare). Equal amounts of total RNA from each sample were subjected to cDNA synthesis using reverse transcriptase (Fermentas Life Sciences). Real-time PCR was performed in an ABI StepOne System (Applied Biosystems) using the SYBR Green detection protocol as outlined by the manufacturer. Samples were examined in triplicate in each experiment and analyzed by using the 2^−ΔΔCT^ method. Expression of GAPDH was used as an internal control for cDNA content. The ΔCT of a target gene after stimulation was compared to the ΔCT of the same target gene before stimulation. The results were presented as fold changes. Gene-specific amplification was conducted with the following primer sets: for delta-aminolevulinate synthase 2 (ALAS2), 5′-GCAGCACTCAACAGCAAG-3′ (sense) and 5′-ACAGGACGGCGACAGAAA-3′ (antisense); for γ-globin, 5′-CCATAAAGCACCTGGATGATC-3′ (sense) and 5′-ATCTGGAGGACAGGGCACTG-3′ (antisense); for porphobilinogen deaminase (PBGD), 5′-CGCCTCCCTCTAGTCTCTGCTTCT-3′ (sense) and 5′- GTTGCCACCACACTGTCCGTCTG-3′ (antisense); for erythroid Krüppel-like factor (EKLF), 5′-CGGCAAGAGCTACACCAAG-3′ (sense) and 5′-CCGTGTGTTTCCGGTAGTG-3′ (antisense); for nuclear factor-erythroid-derived 2 (NF-E2), 5′-ACAGAGAGCCAGCTAGAGCT-3′ (sense) and 5′- TGGGCTGCCACCTTGTTT-3′ (antisense); for GATA1, 5′-CAGTCTTTCAGGTGTACCC-3′ (sense) and 5′-GAGTGATGAAGGCAGTGCAG-3′ (antisense); for β–actin, 5′-TCGTGCGTGACATTAAGGAG-3′ (sense) and 5′-ATGCCAGGGTACATGGTGGT-3′ (antisense).

### *In vitro* Protein Kinase Assay

The p38 kinase assay was performed using a nonradioactive p38 kinase assay kit (Cell Signaling). Briefly, active p38 MAPK was immunoprecipitated with an immobilized anti-phospho-p38 antibody and assayed for kinase activity with ATF2 peptides (1 µg) as the substrate. Reactions were stopped with SDS sample buffer and subjected to Western blotting with a specific anti-phospho-ATF2 antibody according to the manufacturer’s instruction.

### Plasmids and Selection for Stable Clones

K562 cell clones stably expressing HA-RMT1 (R2–1 and R2–3) or HA-PRMT1G80R (G80R-15) from pcDNA3HA2 plasmids were established as previously described [22]. The mammalian p38α and p38β expression plasmids were a gift from Dr. J. Han (The Scripps Research Institute, La Jolla, CA). The pLKO.1-puro plasmid-based shRNAs, including shLuc (luciferase shRNA), PRMT1-sh1, p38α-sh1, p38α-sh2, p38β-sh1, p38β-sh2, and p38β-sh3, were obtained from the National RNAi Core Facility, Taiwan. These plasmids were used to selectively knock down PRMT1, p38α, or p38β genes. Transfection of K562 cells was performed using Lipofectamine™ 2000 Reagent (Invitrogen). Stable clones of PRMT1 KD-1, PRMT1KD-2, p38α KD-1, p38α KD-2, p38β KD-1 and p38β KD-2 were selected with puromycin (0.5 µg/ml) as described [22].

### Expression and Purification of Recombinant Proteins

Recombinant proteins including hnRNPs, TAT-fused HA-PRMT1, TAT-fused GFP, and His-p38α were expressed in *E. coli* BL21(DE3)pLysS, immobilized on Ni^+^-NTA agarose (Qiagen) and eluted with elution buffer (20 mM Tris-HCl, pH 7.9, 0.5 M imidazole, and 500 mM NaCl). The eluted proteins were subjected to dialysis and stored in a buffer containing 20 mM Tris-HCl, pH 7.9, and 50 mM NaCl as described previously [22], [23], [24]. Endotoxins were removed using Detoxi-Gel™ Endotoxin Removing Gel (PIERCE) according to the manufacturer’s instructions.

### Methylation of p38α by PRMT1

Methylation of recombinant His-p38α by GST-PRMT1 was carried out by *in vitro* methylation assay using ^3^H-AdoMet as a methyl donor and detected by fluorography.

### Statistical Analysis

All results shown are representative of three separate experiments. Results are presented as means ± S.E. Statistical comparison between two groups was analyzed using Student’s *t*-test. Values of *p*<0.05 were considered significant. Each differentiation experiment was performed in triplicate and repeated independently for at least three times.

## Results

### Protein Arginine Methyltransferase 1 Enhances Erythroid Differentiation of K562 Cells

**Figure 1:**
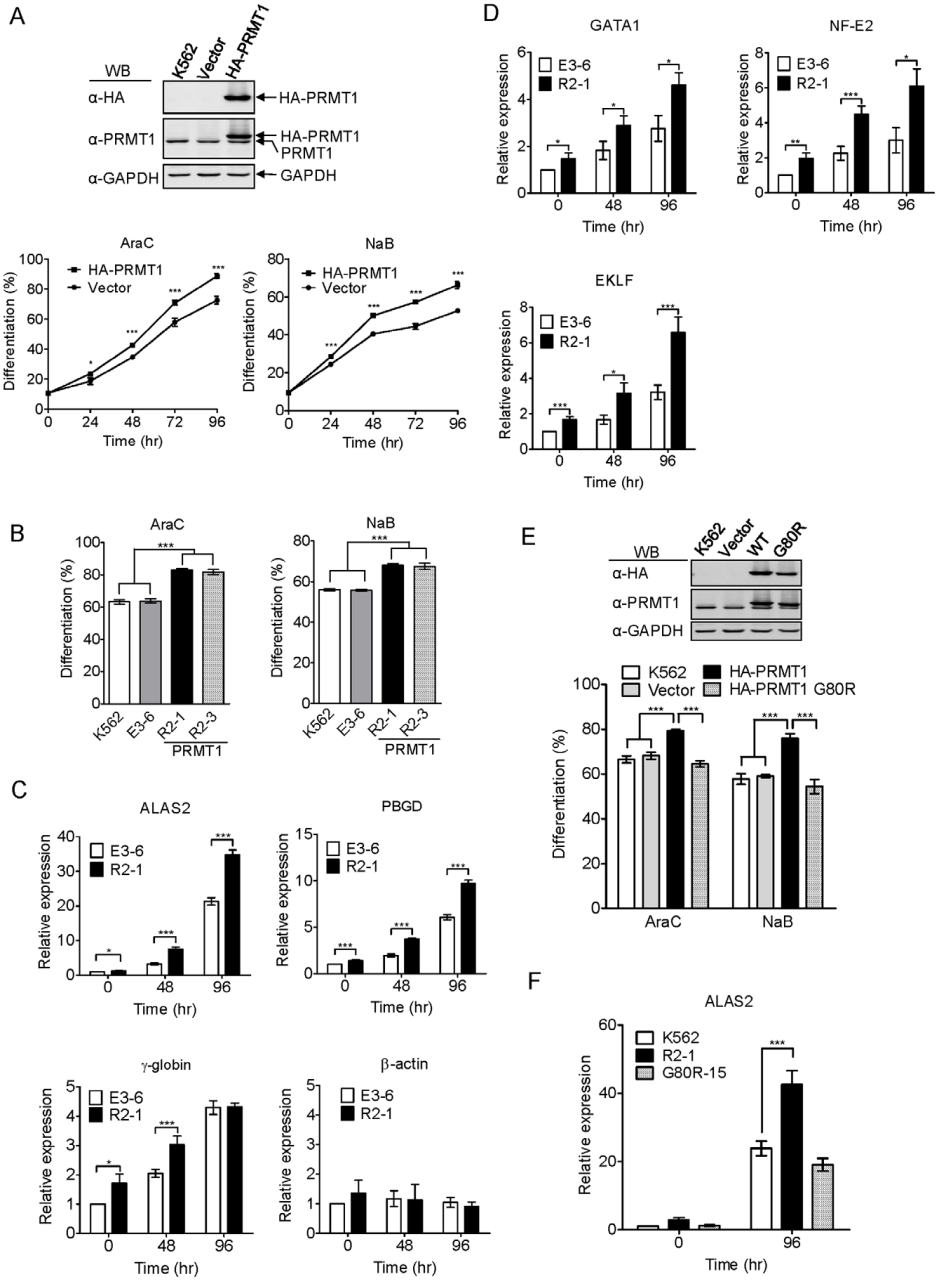
Ectopic expression of PRMT1 enhanced erythroid differentiation of K562 cells. (A) K562 cells were transiently transfected with either empty vectors or pPCDNA3HA2-PRMT1 plasmids expressing HA-PRMT1 and were treated with either araC (1 µM) or NaB (2 mM). Transient expression of PRMT1 significantly enhanced erythroid differentiation, as detected by benzidine staining. (B) R2–1 and R2–3 cells, which stably expressed HA-PRMT1, also exhibited higher erythroid differentiation. E3–6 cells were an empty vector control. (C, D) The expression of various erythroid-related genes and transcription factors was examined by real-time PCR after araC treatment. The β-actin gene was a control whose expression was not affected by araC treatment. Data were calculated as fold changes using vector control cells without treatment as a reference. (E) The enzymatically impaired G80R PRMT1 mutant, when introduced by transient transfection, failed to enhance differentiation. (F) The stable clone that expressed the G80R mutant also failed to stimulate ALAS2 gene expression. All results shown are representative of three separate experiments. Differentiation results are presented as means ± S.E. of three repeats; *, p<0.05; **, p<0.01; ***, p<0.005 compared with vector control cells.

We found in our preliminary examination that the cellular profile of protein arginine methylation significantly changed during erythroid differentiation of K562 cells. Since PRMT1 is the predominant protein arginine methyltransferase, we investigated the role of PRMT1 in erythroid differentiation. K562 cells were transiently transfected with the control vector or pcDNAHA2-PRMT1 to express the N-terminally tagged HA-PRMT1 proteins (Figure 1A, upper panel) and induced for erythroid differentiation. Benzidine staining for hemoglobin showed that overexpression of HA-PRMT1 significantly promoted both araC-induced and sodium butyrate (NaB)-induced erythroid differentiation (Figure 1A, lower panels). K562 cell clones R2–1 and R2–3, which stably expressed HA-PRMT1 [22], also exhibited a higher efficiency in araC or NaB induction of erythroid differentiation, compared to the parental or vector control cells (Figure 1B). The effect of PRMT1 was further examined in a different erythroleukemia cell line HEL. Transient overexpression of HA-PRMT1 also stimulated NaB-induced erythroid differentiation in HEL (Figure S1). These results suggest that the stimulatory impact of PRMT1 is not restricted to K562 cells.

To confirm the effect of PRMT1 expression on erythroid differentiation, we examined the transcripts of γ-globin and two key enzymes involved in heme synthesis, i.e., delta-aminolevulinate synthase 2 (ALAS2) and porphobilinogen deaminase (PBGD). Real-time PCR results showed that araC treatment stimulated the expression of these genes in a time-dependent manner in the vector control cells, and stable expression of PRMT1 indeed significantly increased the transcript levels of ALAS2, PBGD and γ-globin (Figure 1C). GATA1, NF-E2 and EKLF are transcription factors critically involved in the differentiation toward the erythroid lineage [26]. We found that the expression of these three factors in control cells were increased during differentiation and overexpression of PRMT1 significantly enhanced the transcript levels of these transcription factors (Figure 1D). The stimulatory effects were similarly observed in R2–3 cells (Figure S2). These results suggest that PRMT1 may promote the erythroid differentiation program and not just the hemoglobin synthesis.

The mutation of Gly80 to Arg80 impairs the methyltransferase activity of PRMT1 [22]. Transient expression of the PRMT1 G80R mutant (Figure 1E, upper panel) was unable to enhance araC- or NaB-induced erythroid differentiation in K562 cells like the wild type PRMT1 did (Figure 1E, lower panel). The G80R mutant also failed to stimulate ALAS2 gene expression (Figure 1F). These results suggest that the methyltransferase activity of PRMT1 is essential for its stimulatory effect on erythroid differentiation.

### Knockdown of PRMT1 Significantly Decreases Erythroid Differentiation

**Figure 2:**
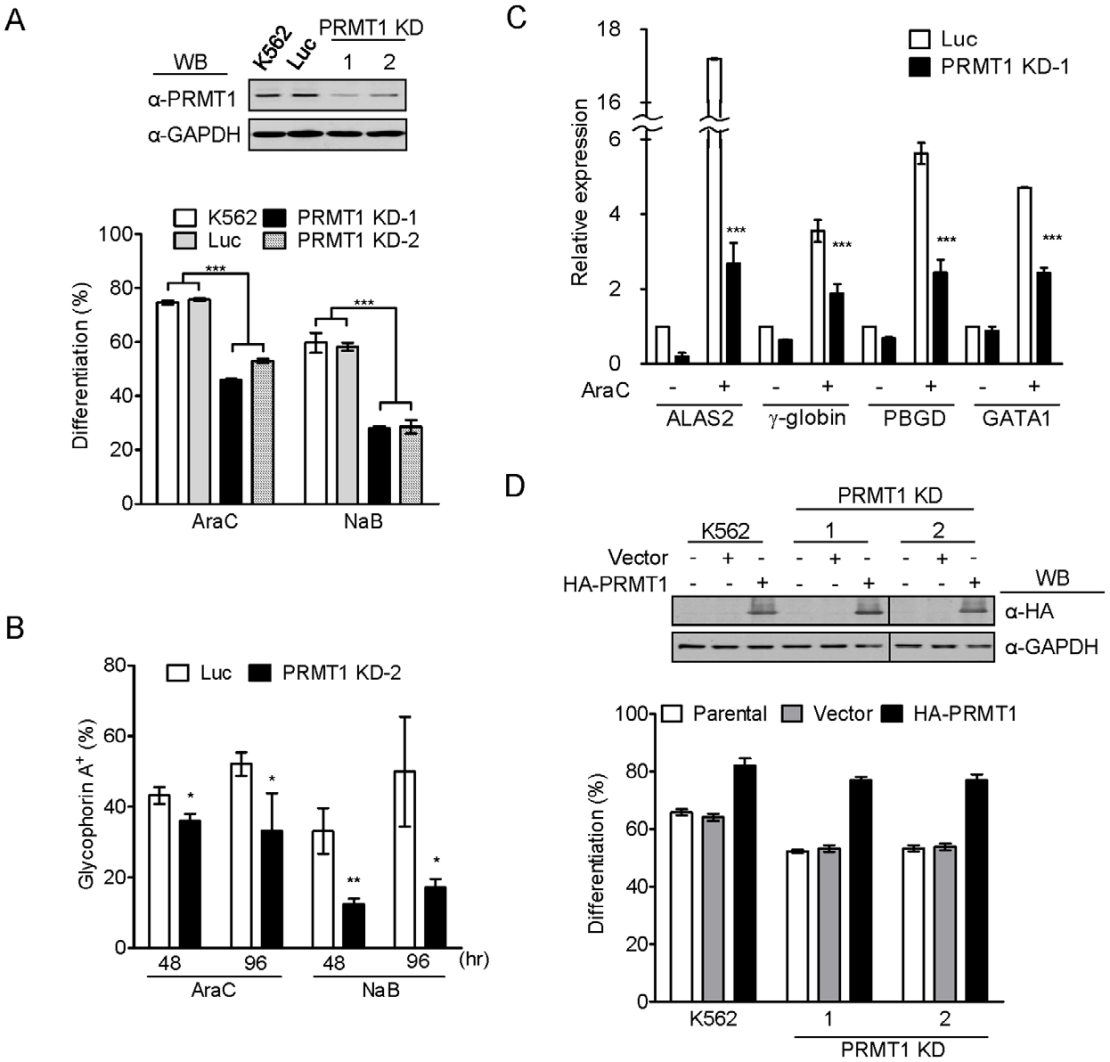
Knockdown of endogenous PRMT1 proteins suppressed erythroid differentiation. (A, upper) Endogenous PRMT1 proteins were greatly reduced in KD-1 and KD-2 cell clones that stably expressed PRMT1 shRNAs. (A, lower, B) Erythroid differentiation was significantly suppressed as detected by benzidine staining or surface expression of glycophorin A at 96 hr after induction. Luc indicates the luciferase shRNA control cells. (C) Gene expression was examined 96 hr after araC treatment by quantitative real-time PCR. Data were calculated as fold changes using vector control cells without treatment as a reference. (D) Re-introduction of HA-PRMT1 into KD-1 and KD-2 cells was performed by transient transfection and the effect on araC-induced differentiation was analyzed after 96 hr. All results shown are representative of three separate experiments. Differentiation results are presented as means ± S.E. of three repeats; *, p<0.05; **, p<0.01; ***, p<0.005 compared with vector control cells.

To examine the role of endogenous PRMT1 in erythroid differentiation, we used PRMT1-deficient clones PRMT1 KD-1 and KD-2 (Figure 2A), which were established previously by selecting clones stably expressing PRMT1 shRNAs [22]. Knocking down endogenous PRMT1 expression suppressed both araC-induced and NaB-induced erythroid differentiation (Figure 2A). Cell-surface expression of glycophorin A, a characteristic marker of erythrocytes, induced by araC and NaB was also suppressed in PRMT1-deficient cells (Figure 2B and Figure S3A). PRMT1-deficient cells also exhibited reduced expression of ALAS2, PBGD, γ-globin and GATA1 transcripts (Figure 2C and Figure S3B). Furthermore, when PRMT1 was re-introduced into PRMT1-deficient cells by transfection, the differentiation of these cells was restored to a level comparable to that in the parental and vector control cells (Figure 2D). These results provide solid evidence that endogenous PRMT1 positively regulates the differentiation toward the erythroid cell lineage.

### The Activity of p38 MAPK is Required for the Effect of PRMT1 on Erythroid Differentiation

**Figure 3:**
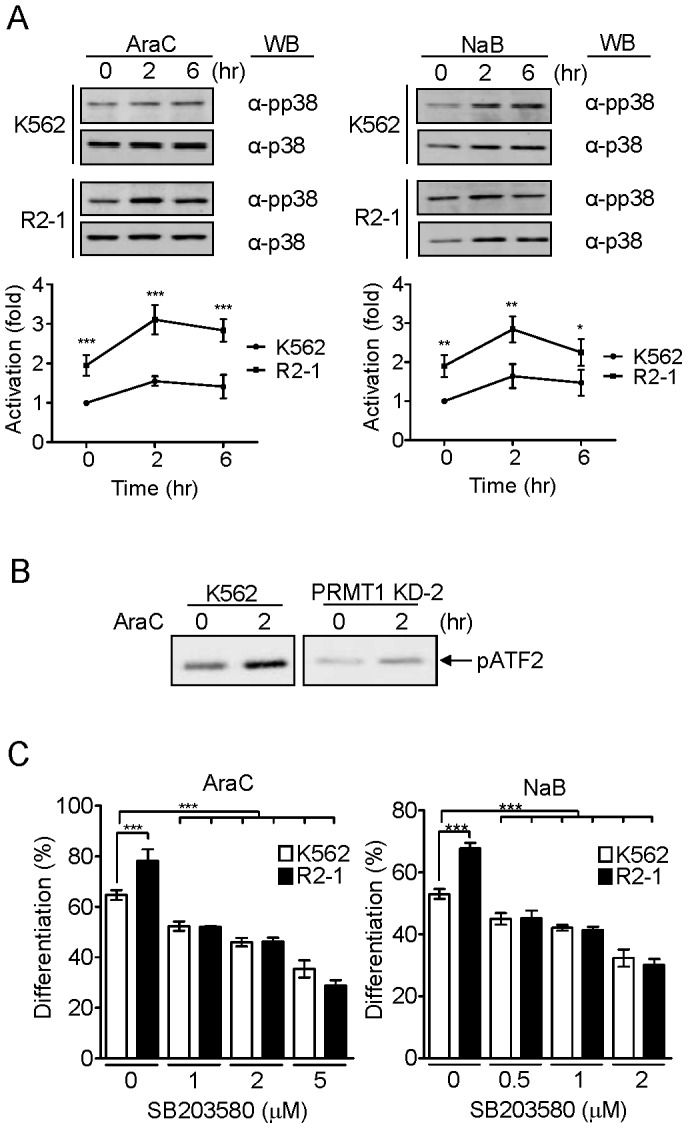
The PRMT1-mediated effect on erythroid differentiation required the activation of p38 MAPK. (A) Phosphorylation of p38 MAPK was stimulated upon araC and NaB treatment in parental K562 cells. Significantly higher levels of p38 MAPK activation were observed in the PRMT1-overexpressing R2–1 cell clone. The levels of phospho-p38 were compared to those detected in control K562 cells without stimulation. Data are presented as means ± S.E. of three independent experiments. (B) In contrast, the activation of p38 MAPK was greatly reduced in PRMT1-knockdown KD-2 cells, as examined by an *in vitro* kinase assay using ATF-2 as the substrate. (C) The pharmacological inhibitor of p38 MAPK, SB203580, inhibited erythroid differentiation of parental K562 cells and R2–1 cells. All results shown are representative of three separate experiments. Differentiation results are presented as means ± S.E. of three repeats; *, p<0.05; **, p<0.01; ***, p<0.005 compared with K562 control cells.

The p38 MAPK pathway has been shown to play a pivotal role in regulating erythroid differentiation [4]. We examined whether PRMT1 potentiated the activation of p38 MAPK. p38 MAPK is activated via dual phosphorylation on specific threonine and tyrosine residues by the upstream MKKs (MAP kinase kinases) [27]. By monitoring the level of phosphorylated p38 using Western blotting, we found that araC and NaB treatment stimulated the activation of p38 MAPK in parental K562 cells, and in the PRMT1-expressing R2–1 clone the activation was significantly enhanced 2 to 3 fold (Figure 3A). Similar results were observed in R2–3 cells (Figure S4A). The Erk1/2 MAPK was not activated by either araC or NaB in parental K562 cells (Figure S4B). Ectopic expression of HA-PRMT1 did not appear to affect activation of Erk1/2 to any significant extents (Figure S4B).

We further performed an *in vitro* kinase assay using ATF2 as the protein substrate, and demonstrated that the kinase activity of p38 MAPK was greatly suppressed in PRMT1-knockdown KD-1 and KD-2 cells (Figure 3B and Figure S4C). The pharmacological inhibitor of p38 MAPK, SB203580, suppressed araC- and NaB-induced erythroid differentiation in a dose-dependent manner in K562 cells, confirming an essential role of p38 MAPK in erythroid differentiation; notably, SB203580 limited the enhancement of differentiation observed in PRMT1-expressing R2–1 cells, suggesting that the differentiation-stimulating effect of PRMT1 expression was dependent on p38 activation (Figure 3C). Similar results were also observed in R2–3 cells (Figure S4D). Together, these results suggest that PRMT1 acts by enhancing the activation of p38 MAPK to promote erythroid differentiation.

### p38α, but not p38β, Mediates the Stimulatory Effect of PRMT1 on Erythroid Differentiation

**Figure 4:**
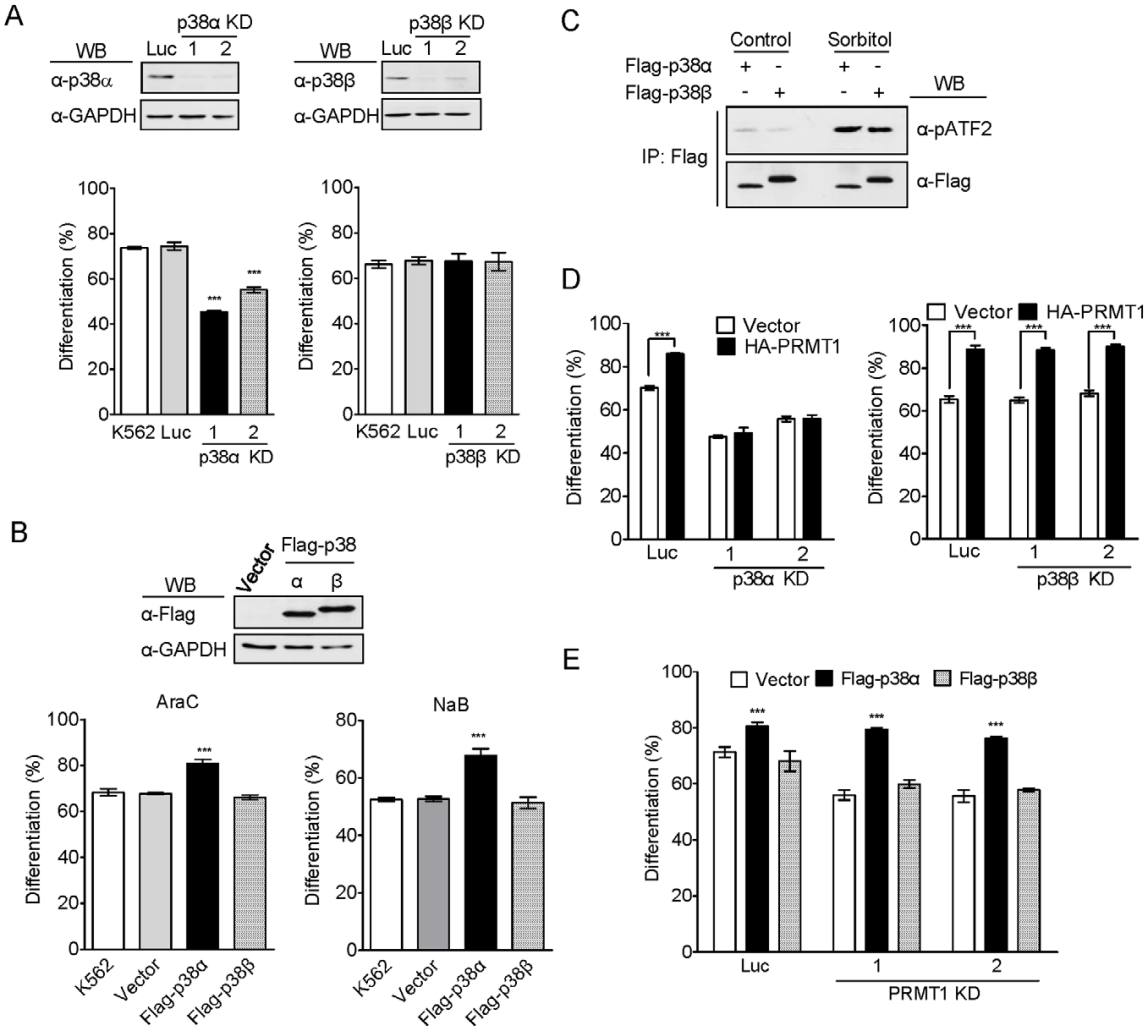
p38α, but not p38β, mediated the stimulatory effect of PRMT1 on erythroid differentiation. (A) Stable expression of shRNAs specific for either p38α or p38β depleted p38α and p38β proteins in K562 cells, respectively. Luc represents the luciferase control cell clone. Knocking down p38α (p38α KD) significantly suppressed araC-induced erythroid differentiation, whereas knockdown of p38β (p38β KD) had no apparent effect. (B) Consistently, ectopic expression of Flag-p38α enhanced erythroid differentiation, whereas Flag-p38β had no effect. (C) The ectopically expressed Flag-p38α and Flag-p38β proteins exhibited similar kinase activity upon sorbitol stimulation, indicating that they are both functionally active enzymes. (D) In p38α KD cell clones, ectopic expression of HA-PRMT1 failed to enhance araC-induced erythroid differentiation; however, HA-PRMT1 still retained its stimulatory effects in p38β KD cell clones. (E) Notably, Flag-p38α effectively stimulated araC-induced erythroid differentiation even in the PRMT1-deficient cell clones KD1 and KD2. All results shown are representative of three separate experiments. Differentiation results are presented as means ± S.E. of three repeats; *, p<0.05; **, p<0.01; ***, p<0.005 compared with control cells.

Although p38 MAPK has been shown to play a crucial role in regulating erythroid differentiation, the role of each isoform has not been fully elucidated. p38α and p38β are the most closely related members [27]. We first selectively knocked down either endogenous p38α or p38β using specific shRNAs; stable cell clones were obtained that showed a significant deficiency in either p38α or p38β proteins (Figure 4A, upper panel). Knockdown of p38α resulted in dramatically reduced erythroid differentiation induced by araC, while knockdown of p38β had no effect (Figure 4A, lower panel), indicating a crucial role of the α but not the β isoform of p38. Consistent with the above finding, expression of Flag-p38α but not Flag-p38β enhanced erythroid differentiation (Figure 4B). *In vitro* kinase assays demonstrated that the Flag-p38α and Flag-p38β proteins expressed in K562 cells were similarly activated when cells were stimulated with sorbitol, a known activator for p38 MAPK [28], indicating that both were functionally active kinases (Figure 4C). These results indicate that p38α plays a positive role in regulating erythroid differentiation, whereas p38β does not participate in this regulation.

To further establish the link between p38α and PRMT1, we transiently expressed HA-PRMT1 in K562-derived cell clones, and found that expression of PRMT1 promoted erythroid differentiation in the control cells but not in the p38α-deficient clones (Figure 4D), suggesting that the stimulatory effect of PRMT1 requires the presence of p38α. Notably, knockdown of p38β did not affect the stimulatory effect of PRMT1 (Figure 4D), confirming that p38β is not involved in PRMT1-mediated modulation of erythroid differentiation. Expression of p38α in PRMT1-knockdown cell clones was still able to enhance erythroid differentiation (Figure 4E), suggesting that p38α acts downstream of PRMT1. Consistently, the expression of p38β did not have apparent effects in either control or PRMT1-knockdown cells (Figure 4E). These results demonstrate that p38α, but not p38β, mediates the stimulatory effect of PRMT1 on erythroid differentiation.

### PRMT1 Binds with and Methylates p38α

**Figure 5:**
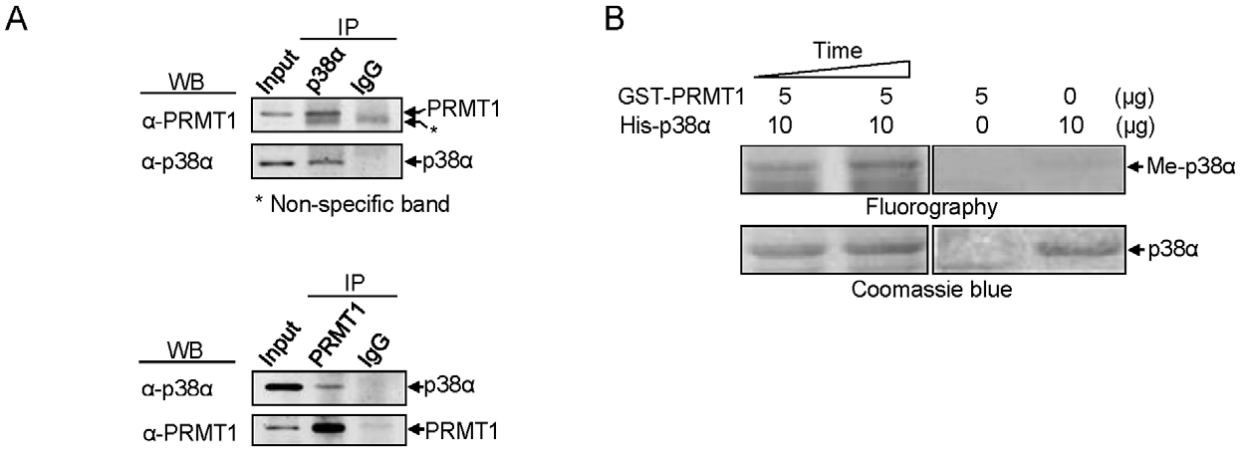
PRMT1 physically interacted with and methylated p38α. (A) An anti-p38α specific antibody was used to immunoprecipitate p38α from K562 cell lysates. PRMT1 was associated with the precipitates (upper panel). In addition, p38α was co-immunoprecipitated when an anti-PRMT1 specific antibody was used (lower panel). Mouse IgG was used as a negative control. (B) The recombinant His-p38α protein was methylated *in vitro* by GST-PRMT1 in a time-dependent manner (30–60 min) as detected by fluorography. No methyl incorporation was observed in the absence of either the substrate (p38α) or the enzyme (PRMT1). All results shown are representative of three separate experiments.

To investigate the possibility that p38α is a direct target of PRMT1, we first performed immunoprecipitation in K562 cells. When p38α was immunoprecipitated using a specific antibody, the endogenous PRMT1 protein was co-precipitated (Figure 5A, upper). Consistently, when PRMT1 was immunoprecipitated, p38α was associated with the precipitates (Figure 5A, lower). These results demonstrate the physical interaction between PRMT1 and p38α, and suggest that PRMT1 may activate the p38α pathway by directly targeting p38α. An *in vitro* methylation assay was carried out to test whether PRMT1 can methylate p38α. The results showed that methyl incorporation into the recombinant p38α proteins increased in a time-dependent manner, while no methylation was detected in the absence of either PRMT1 or p38α (Figure 5B). These results showed for the first time that p38α can serve as a substrate for PRMT1. Together, our results suggest that PRMT1 may enhance the activation of p38α by directly binding to and methylating p38α.

### PRMT1 Enhances the EPO-induced Erythroid Differentiation of Primary Human CD34^+^ Hematopoietic Progenitor Cells through Activation of p38 MAPK

**Figure 6:**
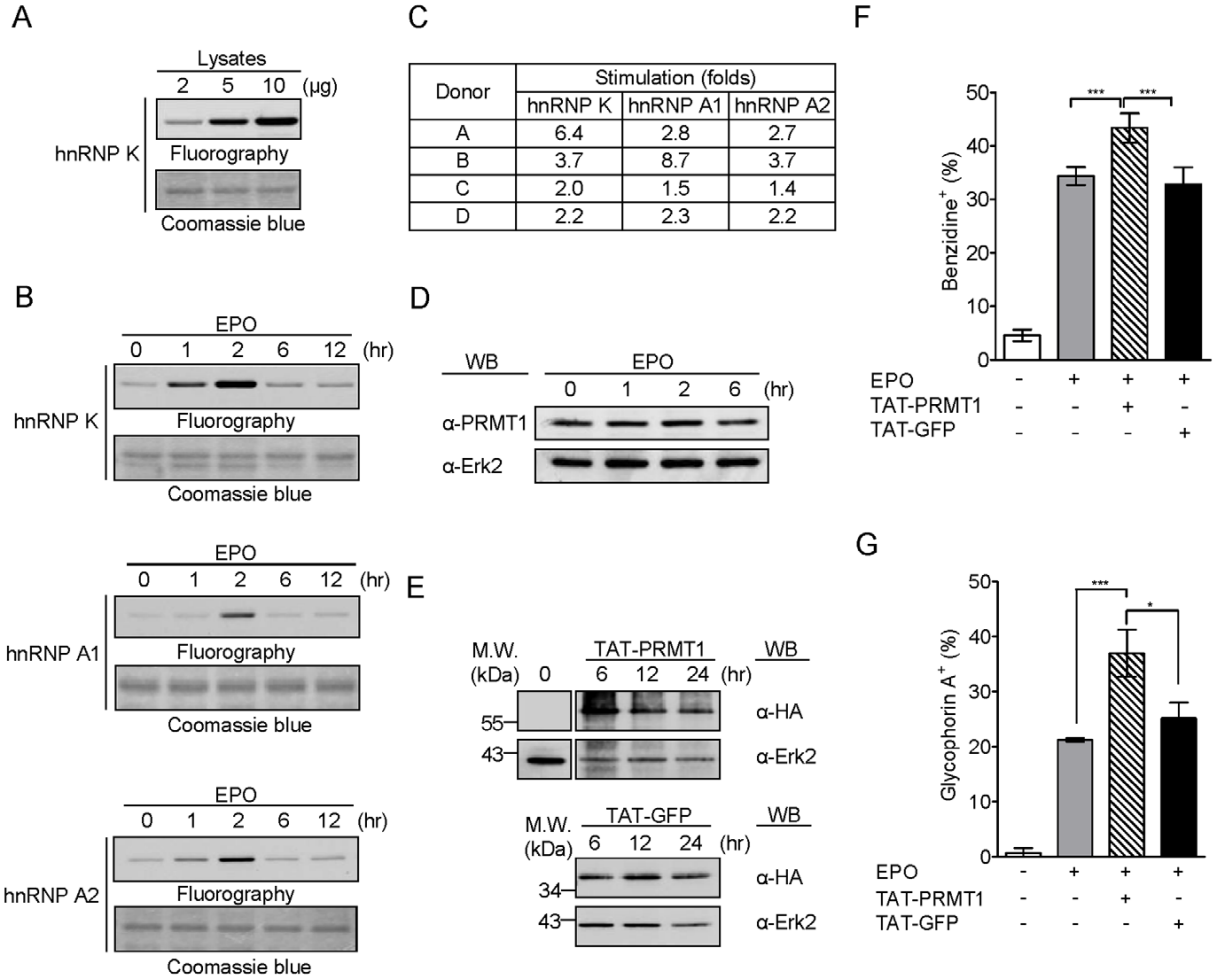
PRMT1 stimulated EPO-induced erythroid differentiation in primary human CD34^+^ hematopoietic progenitor cells which required the activation of p38 MAPK. (A) The PRMT1 methyltransferase activity in the homogenates of human CD34^+^ cells was assayed by *in vitro* methylation using hnRNP K (2 µg) as a substrate and visualized by fluorography. (B) Upon EPO treatment, PRMT1 activity was rapidly stimulated and reached its peak at 2 hr, as shown with three different substrates: hnRNP K, hnRNP A1 and hnRNP A2. A representative fluorograph from Donor A is shown. (C) The results from four individuals were expressed as fold stimulation at 2 hr after EPO treatment. Methyl incorporation into protein substrates was quantified by liquid scintillation counting. (D) PRMT1 protein levels did not change after EPO treatment. (E) The recombinant TAT-conjugated HA-PRMT1 proteins were introduced into CD34^+^ cells through the protein transduction method. HA-GFP recombinant proteins served as a negative control. (F, G) TAT-PRMT1 (0.1 µM) significantly stimulated the EPO-induced erythroid differentiation of CD34^+^ cells, as measured by benzidine staining and the surface expression of glycophorin A. Experiments were performed with cells from at least three different donors. Data are presented as means ± S.E. of at least three independent experiments; *, p<0.05; **, p<0.01; ***, p<0.005 compared with EPO-treated cells.

EPO is the primary cytokine that regulates various steps in the differentiation of erythrocytes in the body [2]. To study the role of PRMT1 in a more physiologically relevant cell model, EPO-induced erythroid differentiation of primary CD34^+^ hematopoietic progenitor cells isolated from human umbilical cord blood was examined. The methyltransferase activity of PRMT1 in CD34^+^ cell homogenates was readily measured using hnRNP K as the substrate; methyl incorporation increased in a dose-dependent manner (Figure 6A). Using three known PRMT1 substrate, hnRNP K, hnRNP A1 and hnRNP A2, in this *in vitro* methylation assay, we showed that upon the EPO treatment of CD34^+^ cells, the PRMT1 activity was rapidly stimulated and reached its peak at 2 hr (Figure 6B). Considering individual variations, we performed assays on CD34^+^ cells isolated from different donors and quantified the methylation of three PRMT1 substrates 2 hr after EPO treatment; the results showed that homogenates from all donors contained an EPO-induced methyltransferase activity, although the fold of stimulation varied (Figure 6C). While the methyltransferase activity in CD34^+^ cells was stimulated by EPO, the protein level remained stable (Figure 6D), suggesting that the activity of PRMT1 may be regulated in response to EPO signaling. To evaluate the role of PRMT1, we then introduced PRMT1 into CD34^+^ progenitor cells by the TAT-mediated protein transduction method [22], [29]. The TAT-fused PRMT1 and the control TAT-GFP proteins entered the cells rapidly and remained in the cells for up to 24 hr (Figure 6E). TAT-PRMT1 significantly stimulated EPO-induced erythroid differentiation, as measured by either benzidine staining (Figure 6F) or surface expression analysis of glycophorin A (Figure 6G). Taken together, our results demonstrate that PRMT1 plays a positive role in modulating erythroid differentiation not only in K562 cells but also in human primary CD34^+^ hematopoietic progenitor cells induced by EPO.

## Discussion

Despite intensive studies, the molecular events that regulate erythropoiesis are not fully elucidated. This study reveals for the first time that PRMT1, the predominant enzyme responsible for cellular protein arginine methylation, plays a crucial role in promoting erythroid differentiation in bipotent leukemia cells as well as in human primary CD34^+^ hematopoietic progenitor cells. Moreover, we have identified a specific isoform of p38 MAPK, p38α, that acts downstream of PRMT1 in mediating the stimulatory effect on erythroid differentiation. Furthermore, we show that PRMT1 is associated with p38α in cells and demonstrate that p38α is a direct substrate for PRMT1 *in vitro*. These results highlight a novel regulatory mechanism involving protein arginine methylation in modulating the signaling pathways leading to the development of the erythroid lineage.

A number of studies have reported that p38 MAPK is involved in erythroid differentiation [6], [7], [8], yet the upstream regulatory events required for the activation of p38 MAPK in this developmental program are still poorly understood. The p38 MAPKs are modulated by a phosphorylation cascade which leads to dual phosphorylation on threonine/tyrosine residues and the activation of the catalytic activity [27]. Dephosphorylation of MAPKs by specific phosphatases turns off the signaling event [30]. In addition, the MAPK signaling complex binds to scaffold proteins, which regulates the specificity of downstream events by influencing the enzyme’s location, catalytic activity, duration of activation and substrate selection [27]. The association between PRMT1 and p38α discovered in this study suggests another potential regulatory mechanism for MAPK signaling. It is conceivable that methylation of p38α by PRMT1 may make it a better substrate for MAP kinase kinases (MKKs), reduce its dephosphorylation, or affect its interaction with scaffold or other regulatory proteins.

Methylation by PRMT1 produces mono- and asymmetric dimethyl- arginines which has been reported to affect phosphorylation through various mechanisms. When occurs in the consensus motif for Akt-mediated phosphorylation (RxRxxS/T), arginine methylation of FOXO1 transcription factor and BAD (BCL-2 antagonist of cell death) counteracts their phosphorylation by Akt and abrogates Akt-mediated signaling [18], [19]. Arginine methylation may provide the binding surface for interacting partners and thus positively or negatively regulates phosphorylation events. PRMT1-mediated methylation of ASK1 (apoptosis-signal-regulating kinase 1) promotes its interaction with the negative regulator thioredoxin and thus blocks the H_2_O_2_-induced activation of this kinase [31]. Methylation of Axin, a regulator of Wnt singaling, by PRMT1 promotes its interaction with and phosphorylation by glycogen synthase kinase 3β which leads to stabilization of Axin [21]. Further investigation is required to determine the effects of arginine methylation of p38α at the molecular level.

PRMT1 may have multiple targets in mediating the regulation of erythropoiesis. Although we have demonstrated that p38α is a PRMT1 substrate, given the scaffolding mechanism in MAPK signaling, we cannot rule out the possibility that PRMT1 also interacts with other components in the p38 signaling pathway. Moreover, our results show that PRMT1 promotes multiple aspects of the erythroid differentiation program, including the characteristic erythroid cell phenotypes and the expression of transcriptional regulators critical for lineage-specific differentiation. In addition to genes encoding γ-globin and two key enzymes for heme synthesis, transcription factors GATA1, NF-E2 and ELKF are also up-regulated under PRMT1 overexpression. As both NF-E2 and ELKF are known downstream targets of GATA1 [32], [33], this raises the possibility that GATA1 may be the primary target transcription factor regulated by the PRMT1/p38α pathway. One of the known mechanisms through which PRMT1 activates transcription is by methylating the histone H4 on Arg3, which permits the subsequent acetylation of histones. This is demonstrated in the retinoic acid-induced expression of transglutaminase type 2 (TGM2) gene [34] and the DMSO-induced expression of β-globin gene [16]. The possibility that PRMT1-mediated up-regulation of transcription during erythroid differentiation involves histone H4 Arg3 methylation is not ruled out.

Our analysis of CD34^+^ hematopoietic progenitor cells shows that despite the increase in the methyltransferase activity following EPO treatment, the PRMT1 protein levels maintain steady, suggesting that the enzymatic activity of PRMT1 is regulated during erythroid differentiation. Up-regulation of PRMT1 activity has been reported in NGF-stimulated PC12 cells [35], during fatal liver development, and in the early stage during liver regeneration [36]. The molecular events regulating PRMT1 activity during these cellular processes are not clear. The activity of PRMT1 can be regulated by protein interactions. The interacting proteins, TIS21/BTG2 and BTG1, have been shown to modulate the substrate selectivity of PRMT1 [12]. CCR4-associated factor 1 (hCAF1) regulates PRMT1 activity in a substrate-dependent manner [37]. TR3, an orphan receptor, causes an inhibition of the methyltransferase activity of PRMT1 by directly binding to the catalytic domain [38]. In addition, when phosphorylated, the methyltransferase activity of CARM1 (PRMT4) and PRMT5 is inhibited [39], [40]. It is not yet clear whether the enzymatic activity of PRMT1 is regulated by posttranslational modifications.

Erythroid cells and megakaryocytes share common bipotent megakaryocyte/erythrocyte progenitors [41]. Differentiation of progenitors toward these two lineages is mutually exclusive. The molecular mechanisms controlling this lineage-specific differentiation have only been partially elucidated. Several molecules are reported to serve as a switch in governing commitment/differentiation toward specific lineages. A deficiency of erythroid Krüppel-like factor (EKLF) enhances the formation of megakaryocytes and suppresses erythroid cells [42], [43]. c-Myc^−/−^ mice display significantly increased numbers of megakaryocytic progenitors and a blockage of erythroid progenitors [44]. This study unveils a novel link between PRMT1 and p38 in positively regulating the erythroid differentiation program and suggests a novel regulatory mechanism for p38α through arginine methylation. Our previous study has shown that PRMT1 plays a negative role in megakaryocytic differentiation [22]. Taken together, these studies suggest that PRMT1, mediated by p38α, serves as a novel molecular switch in lineage-specific differentiation toward megakaryocytes or erythrocytes. Our results have significant impacts on our understanding of how protein arginine methylation mediated by PRMT1 and protein phosphorylation mediated by p38α are coordinated in regulating hematopoietic differentiation. These findings also suggest that PRMT1 and p38α can potentially serve as novel targets for therapeutic purposes.

## Supporting Information

Figure S1 **Ectopic expression of PRMT1 enhanced erythroid differentiation of HEL cells.** Cells were transiently transfected with either empty vectors or pPCDNA3HA2-PRMT1 plasmids expressing HA-PRMT1 and treated with NaB (0.1 mM). Transient expression of PRMT1 significantly enhanced erythroid differentiation, as detected by benzidine staining at 24 hr after induction. All results shown are representative of three separate experiments. Differentiation results are presented as means ± S.E. of three repeats; *, p<0.05; **, p<0.01; ***, p<0.005 compared with vector control cells.
(TIF)

Figure S2 **The expression of erythroid-related genes was enhanced in PRMT1-overexpressing R2–3 cells.** The expression of various erythroid-related genes (A) and transcription factors (B) in PRMT1-overexpressing R2–3 cells was examined by real-time PCR after araC treatment. Data were calculated as fold changes using vector control cells without treatment as a reference. All results shown are representative of three separate experiments. Results are presented as means ± S.E. of three repeats. *, p<0.05; **, p<0.01; ***, p<0.005 compared with vector control cells.
(TIFF)

Figure S3 **Knockdown of PRMT1 suppressed the expression of erythroid surface marker glycophorin A and erythroid-related genes.** (A) The surface expression of glycophorin A after stimulation by either araC or NaB was significantly suppressed in PRMT1 KD-1 cells as compared to the vector control cell. (B) Gene expression in PRMT1 KD-2 cells was examined 96 hr after araC treatment by quantitative real-time PCR. Knockdown of PRMT1 significantly suppressed the expression of erythroid-related genes. Data were calculated as fold changes using vector control cells without treatment as a reference. All results shown are representative of three separate experiments. Results are presented as means ± S.E. of three repeats. *, p<0.05; **, p<0.01; ***, p<0.005 compared with vector control cells.
(TIF)

Figure S4 **PRMT1 enhanced the activation of p38 MAPK.** (A) Phosphorylation of p38 MAPK was significantly enhanced in the PRMT1-overexpressing R2–3 cell clone upon araC and NaB stimulation as compared to the control cells. Ratio was determined by using control cells without stimulation as a reference. (B) Stimulation by either araC or NaB did not activate Erk1/2 MAPK in either control cells or PRMT1-overexpressing R2–1 and R2–3 cells. (C) The kinase activity of p38 was significantly reduced in PRMT1-knockdown KD-1 cells when assayed with ATF2 as the substrate. (D) The stimulatory effect of PRMT1-overexpressing R2–3 cells was suppressed by SB203580 to an extent similar to the control cells. All results shown are representative of three separate experiments. Differentiation results are presented as means ± S.E. of three repeats; *, p<0.05; **, p<0.01; ***, p<0.005 compared with control cells.
(TIF)
