# Supplementary figures and images for: Protein Arginine Methyltransferase 1 Interacts with and Activates p38α to Facilitate Erythroid Differentiation
Source: PLoS One. 2013 Mar 6;8(3):e56715. doi: 10.1371/journal.pone.0056715 (PMC3590204; doi:10.1371/journal.pone.0056715)

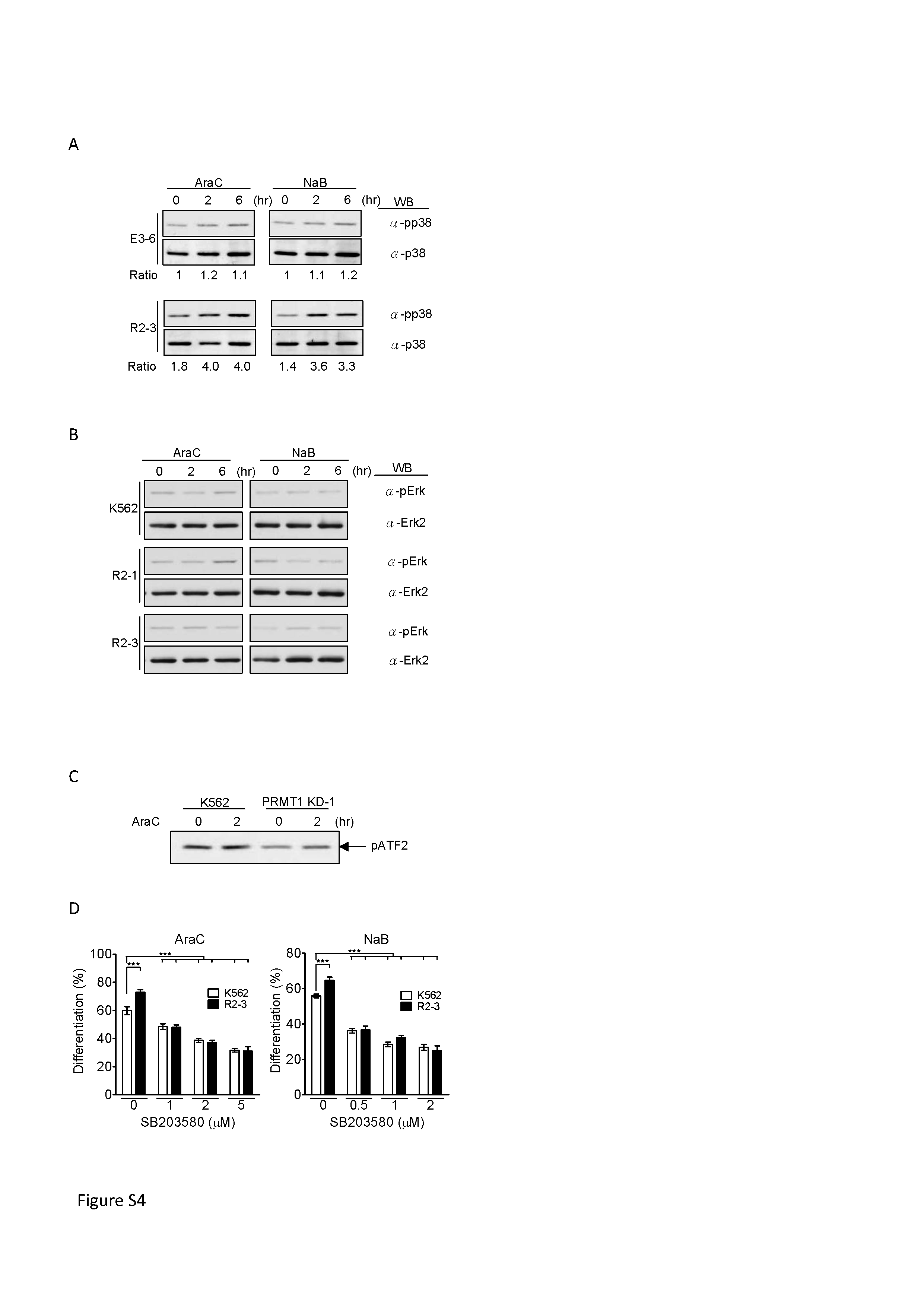

Supplement: Figure S4 — PRMT1 enhanced the activation of p38 MAPK. (A) Phosphorylation of p38 MAPK was significantly enhanced in the PRMT1-overexpressing R2–3 cell clone upon araC and NaB stimulation as compared to the control cells. Ratio was determined by using control cells without stimulation as a reference. (B) Stimulation by either araC or NaB did not activate Erk1/2 MAPK in either control cells or PRMT1-overexpressing R2–1 and R2–3 cells. (C) The kinase activity of p38 was significantly reduced in PRMT1-knockdown KD-1 cells when assayed with ATF2 as the substrate. (D) The stimulatory effect of PRMT1-overexpressing R2–3 cells was suppressed by SB203580 to an extent similar to the control cells. All results shown are representative of three separate experiments. Differentiation results are presented as means ± S.E. of three repeats; *, p<0.05; **, p<0.01; ***, p<0.005 compared with control cells. (TIF) [file pone.0056715.s004.tif]
